# Impact of Chromosomal Inversions on the Yeast DAL Cluster

**DOI:** 10.1371/journal.pone.0042022

**Published:** 2012-08-14

**Authors:** Samina Naseeb, Daniela Delneri

**Affiliations:** Faculty of Life Sciences, University of Manchester, Manchester, United Kingdom; University of Cambridge, United Kingdom

## Abstract

Chromosomal rearrangements occur readily in nature and are a major reshaping force during genome evolution. Such large scale modifications are usually deleterious causing several fitness defects, but sometimes can confer an advantage and become adaptive. For example the DAL metabolic cluster in yeast was assembled in recent evolutionary times in the *Hemiascomycetes* lineage, through a set of rearrangements that brought together the genes involved in the allantoin degradation pathway. In eukaryotes, the existence of physical clustering of genes with related functions supports the notion that neighbouring ORFs tend to be co-expressed and that the order of genes along the chromosomes may have biological significance, rather than being random as previously believed. In this study, we investigate the phenotypic effect that inversions have on the DAL gene cluster, expressed during nitrogen starvation. In all *Saccharomyces* “sensu stricto” species the order of the DAL cluster is conserved, while in the “sensu lato” species *Naumovia castellii*, which grows significantly worse than *S. cerevisiae* on allantoin, the cluster includes two nested inversions encompassing three DAL genes. We constructed several inverted and non-inverted *S. cerevisiae* strains possessing different inversions including those to mimic the configuration of the *N. castellii* DAL cluster. We showed that the inversion of *DAL2* lower its own expression and reduces yeast fitness during nitrogen starvation. This rearrangement also altered the expression of the neighbouring genes *DAL1* and *DAL4*. Moreover, we showed that the expression of the *DAL4* anti-sense transcript (*SUT614*) does not change upon inversions of *DAL2* and therefore is unlikely to be involved in its regulation. These results show that the order of the DAL cluster has an impact on the phenotype and gene expression, suggesting that these rearrangements may have been adaptive in the “sensu stricto” group in relation to the low availability of nitrogen in the environment.

## Introduction

The first evidence of chromosomal inversions was published in 1921 by Sturtevant who studied rearrangement of genes in Drosophila [1]. The current literature shows that inversions have been found in almost all the organisms ranging from prokaryotes to eukaryotes and its rate vary among different lineages [2], [3], [4], [5], [6]. Chromosomal inversions in yeast are predominantly small sometimes including only one gene and can generate a new gene order putting the relevant loci in proximity. Such small chromosomal inversions may be partially responsible for the separation of *Candida albicans* and *Saccharomyces cerevisiae*, since 1,100 single-gene inversions have occurred since the divergence of the two species [7].

It is important to study inversions as they can affect the gene expression of the inverted genes as well as of the neighbouring genes. Genome studies provide an increasing evidence that expression and regulation of genes is not only controlled independently by its own promoters and associated regulatory elements but is also dependent on its location in the genome [8], [9], [10]. It has been indicated that similarly expressed genes are clustered in genomic neighbourhood in almost all taxa, however, it is so far not clear what mechanism enforces them to be clustered or co-localized in the genome. In prokaryotes cluster of genes form operons leading to a strong co-expression and in eukaryotes physically linked genes are co-expressed. The formation of co-expressed clusters during the period of evolution further promotes the fact that gene expression is dependent on its position in the genome [11], [12], [13], [14]. In eukaryotes, it has been argued that co-regulation may not be needed for the physical clustering of genes, and that evolutionary pressure could be sufficient to bring functional genes together [15], [16].

In yeast, *Saccharomyces cerevisiae*, the genes involved in the mitotic cell cycle were the first to be shown as co-expressed clusters in the genome [17]. The size of co-expressed clusters in yeast is relatively small, not exceeding ten genes or few kilobases compared to the clusters of multicellular eukaryotes consisting of 20–30 genes [18].

**Figure 1:**
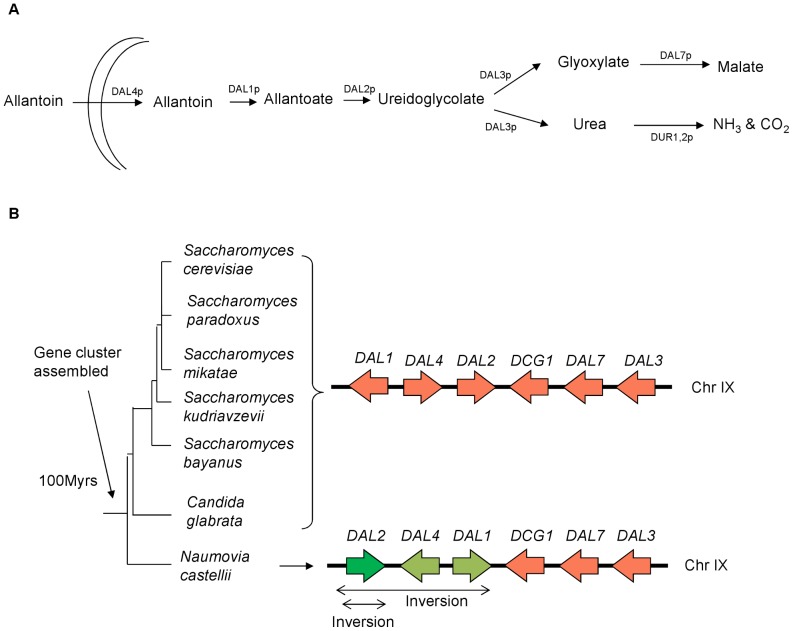
Schematic representation of the yeast DAL gene cluster involved in the allantoin degradation pathway. The panel **A** shows the six DAL genes located on chromosome IX at the same position and orientation in all *S. cerevisiae* “sensu stricto” species. In *N. castellii* the cluster presents two nested inversions involving *DAL1, DAL2* and *DAL4* (marked with a double ended arrow). The colour orange indicates collinear genes, the pale green arrows shows genes which underwent one inversion event and the dark green colour indicate the *DAL2* gene which inverted twice in *N. castellii*. (Figure adapted from Wolf, 2006). The panel **B** shows how allantoin is converted to allontoate by allantoinase and degraded to produce glyoxalate and urea. In the final stage of the pathway, the glyoxalte is converted to malate and the urea to ammonia.

There are two large groups of gene clusters in *S. cerevisiae*, the GAL cluster [19] and the DAL cluster [20]. The DAL cluster is the largest metabolic gene cluster, enabling yeast to use allantoin as a non-preferred nitrogen source. Allantoin is converted through a series of steps into ammonia which is a simpler form of nitrogen readily used by yeast (Figure 1A). The genes in this cluster were initially scattered throughout the yeast genome and co-localised only recently within a single sub-telomeric site on chromosome IX in the ancestor of *S. cerevisiae* and *N. castellii*. The DAL cluster in *S. cerevisiae* consists of six adjacent genes encoding for six of the eight proteins involved in allantoin degradation. While the gene order of the DAL cluster is completely conserved in the *Saccharomyces* “sensu stricto” species, in *N. castellii*, belonging to the “sensu lato” group, the cluster includes two nested inversions (Figure 1B). Other more distantly related hemiascomycetes species still maintain the DAL genes on different chromosomes. *DAL5* (located on chromosome X and encodes for allantoate permease) and *DUR1,2* (located on chromosome II and encodes for a protein which is responsible for converting urea to ammonia) are the two genes which are not located in the DAL cluster [21].

Computational studies provide us with the evidence that species belonging to the *Saccharomyces* “sensu stricto” group present different orientation of the DAL genes compared to *N. castellii* [21]. However, the potential role of these inversions on the phenotype has not been tested experimentally. In this study, we set to investigate the effects of different inversions of the DAL cluster on the gene expression and yeast growth. We constructed various inverted and non-inverted control strains possessing single, double or triple gene inversion as well as a strain resembling the *N. castellii* DAL structure in *S. cerevisiae* background.

We analyzed the fitness of the wild type, inverted and non-inverted control strains in allantoin containing medium that is responsible for triggering the expression of the DAL genes. We showed that the strain mimicking *N. castellii* DAL cluster possessed a lower growth rate as compared to the non-inverted control and the wild type strains. Moreover, we showed that the *DAL2* inversion alone was responsible for the fitness impairment. Gene expression data indicated that *DAL4* gene was down regulated the most in the *DAL2* inverted strain background while the *DAL4* antisense level did not change. Overall, our results indicate that the *DAL2* inversion present in *N. castellii* has a deleterious effect on the growth of *S. cerevisiae* in nitrogen poor medium and reduces the transcript level of *DAL2*, *DAL4* and *DAL1*. As *N. castellii* is growing significantly worse than *S. cerevisiae* in allantoin medium, our results suggests that the rearrangements of the DAL cluster in the *S. cerevisiae* “sensu stricto” species may have conferred some advantage in natural environment knowingly low in nitrogen.

## Materials and Methods

### Strains and Media

All strains were engineered with the FY3 background and they were maintained on YPD medium containing 2% (w/v) yeast extract, 1% (w/v) peptone and 2% (w/v) glucose. The transformants were grown on YPD-agar containing the desired antibiotics i.e. 300 µg/ml geneticin (GibcoBRL), 100 µg/ml cloNAT (Werner BioAgents, Jena, Germany), 10 µg/ml phleomycin (InvivoGen) and 300 µg/ml hygromycin B (Duchefa Biochemie) for selection of the *kanMX*, *natNT2*, *pCre-ble* and *hphNT1* markers. A full list of engineered strains is provided in Table S5. Mineral salt medium (F1 medium) was prepared as previously described [22]. 0.0125% (w/v) allantoin and 0.1% (w/v) proline+0.0125% (w/v) allantoin were used as nitrogen source.

### Primers and Oligonucleotide probes

Gene sequences were obtained from SGD (http://www.yeastgenome.org/) and PCR primers were designed using the Primer3 programme. Strand specific oligonucleotide probes were manually designed keeping the GC content to 40% and length to 34–40 bp. The BLAST tool of SGD was used to check the specificity of each probe and primer. Primer and probe sequences are provided in Tables S1, S2, S3, S4.

### Construction of Inverted and Non-inverted strains

The resistance gene marker cassettes used in this study were *loxP-kanMX-loxP* [23], *loxP-hphNT1-loxP* and *lox2272*-*natNT2-lox2272* [24]. The *loxP-kanMX-loxP* cassette was amplified according to Delneri *et al*. 2003 [25] and *loxP-hphNT1-loxP* and *lox2272*-*natNT2-lox2272* cassettes were amplified according to Janke *et al*. 2004 [26]. These cassettes were inserted in the genome of *S. cerevisiae* by PCR-mediated gene replacement method to construct our inverted and non-inverted strains [27]. Transformation was done by lithium acetate protocol [28]. The strains bearing cassettes were then transformed with Cre-recombinase containing plasmid. The Cre-recombinase enzyme was induced by first growing the cells overnight in YP-raffinose medium and then in YP-galactose for 2–3 hrs. The colonies were verified for inversion and non-inversion by colony PCR [25]. All the primers used for construction of strains are provided in Table S1 and S4.

### Fitness Growth Rate Assay

Growth rate of all the inverted and non-inverted strains was determined using FLUOstar optima microplate reader. Cells were grown to stationary phase in YPD, minimal and allantoin containing medium. Optical density (OD) of the cultures was measured at 595 nm and then diluted to OD_595 nm_ = 0.1 in pre-warmed respective medium. 240 µl of the diluted cultures was transferred to each well of 96 well plate including the media controls. The OD measurement was taken by the microplate reader at 30°C for 40 hours at intervals of 5 minute and with 1 minute linear shaking just before every measurement. Growth curves were plotted using the Optima data analysis programme and standard deviations were calculated in Excel. The growth parameters (growth rate μ and maximum cell biomass *A*) were measured using R statistic package grofit [29].

### RNA extraction and reverse transcription

Total RNA for reverse transcription was extracted using the Qiagen RNA extraction kit following the manufacturer's instructions. RNA concentration was determined using the nanodrop spectrophotometer (ND-1000), quality and integrity of RNA was checked by electrophoresis on 1.5% (w/v) agarose gel. Whenever required RNA was treated with DNaseI (Fermentas) prior to cDNA synthesis as described by the manufacturer. 1 µg of total RNA was reverse transcribed to cDNA in a 20 µl reaction mixture by Qiagen reverse transcription kit using the random primers.

### Gene expression analysis by real-time quantitative PCR

The expression levels of *DAL1*, *DAL2* and *DAL4* were determined using real-time PCR. All real time PCRs were performed on the cDNA samples using the Quantitect real time PCR kit from Qiagen on Chromo4 gradient thermocycler in 96 well plate from Biorad. Real time PCR primers were synthesiszed by MWG-Eurofins (HPSF purified). Each primer was designed to amplify a 250–300 bp fragment. Optimized reactions were carried out in 50 µl final volume containing 10 ng/µl of cDNA, 5 pmole of each primer and 25 µl of 2× quantitect syber green. The qPCR conditions were used with an initial denaturation of 3 min at 95°C followed by 35 cycles consisting of 95°C for 45 sec, 58°C for 45 sec and 72°C for 3 min with a final extension of 5 min at 72°C. Melting curves were analyzed from 55°C to 95°C at a rate of 0.2°/2 sec. Actin (*ACT1*) was used as a housekeeping reference gene. Serial dilutions (10^−1^–10^−5^) of actin DNA was used for generating standard curve. The expression of each gene was estimated using the Ct Values. All real time PCRs were tested in triplicate and each experiment was done on three independent biological replicas. The overall standard deviation is shown in results. A blank (with no RT) was also included in each experiment.

### Northern hybridization

Total RNA for northern blotting was extracted from yeast strains using Trizol (Invitrogen, catalogue # 155-96-018) as described by the manufacturer. 20 µg of the total RNA was loaded in each slot and resolved on 1% (w/v) formaldehyde agarose gel [30]. Samples were prepared by adding RNA loading dye from Fermentas which contained ethiduim bromide. The RNA was transferred on the nylon membrane with panther semidry electroblotter HEP-1 in accordance to the manufacturer's manual using 1XTBE as transfer buffer. The RNA was fixed on the membrane by UV irradiation for 1 min using the UV crosslinker (XL-1500) 1200 µJ/cm^2^ for 60 sec. 5 pmole oligo was labelled with [32p] ATP using T4 polynucleotide kinase (Fermentas, catalogue #EK003). A mixture of three and five oligonucleotide probes binding at different regions in the gene were used for Actin and *DAL4* respectively. The membrane was hybridized at 37°C in oligo hyb solution (0.17Moles Na_2_HPO_4_, 0.079Moles NaH_2_PO_4_, 35g SDS, 1 ml 0.5 M EDTA, dH_2_O to 500 ml and warm to dissolve, pH ∼7.2) for overnight. The membranes were washed at 42°C in 6×SSC for 10 min and 2×SSC, 0.1% SDS for 10 min. The final washing was done at room temperature in 6×SSC. Membranes were exposed to phosphoimager screen for 1–3 days and band intensities were quantified using Quantitect programme from Biorad.

## Results and Discussion

### DAL cluster in *S. cerevisiae* and *N. castellii*

**Figure 2:**
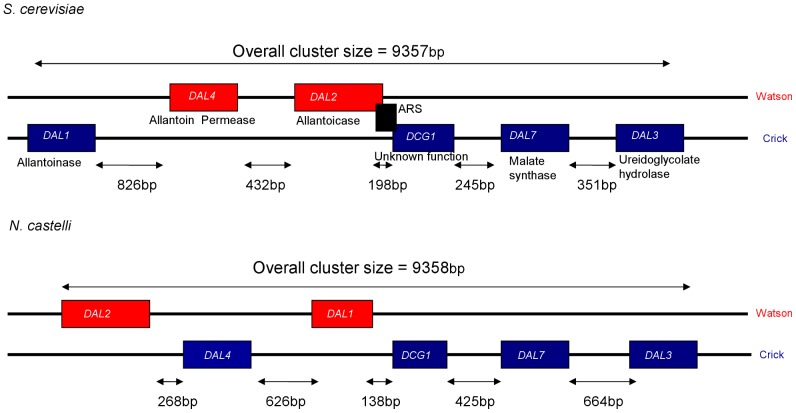
Structure of the DAL cluster. A schematic representation of the size of the DAL cluster and the intergenic regions of *S. cerevisiae* and *N. castellii*. The genes located on the watson and crick strand are coloured red and blue respectively.

*Naumovia castellii* DAL cluster differs from *Saccharomyces cerevisiae* and other sensu stricto species by two nested gene inversion. We compared the sizes of the DAL cluster and that of the intergenic regions between *S. cerevisiae* and *N. castellii* (Figure 2). The overall size of the cluster was almost identical (only 1 base pair different) while the length of the intergenic regions seemed to vary between the two species. We also observed that the sizes of *DAL1* and *DAL3* differ significantly between *S. cerevisiae* and *N. castellii*. *DAL1* is 7% longer in *N. castellii* than in *S. cerevisiae* and *DAL3* is 29% smaller in *N. castellii* than in *S. cerevisiae*. Moreover, in *S. cerevisiae* there is an *ARS* sequence between *DAL2* and *DCG1*. We did not find any *ARS* sequence in *N. castellii* DAL cluster.

### Growth assays of *S. cerevisiae* and *N. castellii* in allantoin medium

The DAL cluster is expressed under nitrogen limited conditions to degrade allantoin (non-preferable source of nitrogen) to a simpler form of nitrogen such as ammonia. Previous literature have shown that *DAL4* mutants grow normally in medium containing either ammonia, allantoate, arginine and asparagines as nitrogen sources, however, they are incapable to grow on allantoin containing medium [31].

Allantoin is a purine derivative formed by oxidation of xanthine to urate and then to allantoin in purine degradation pathway. This reaction is catalyzed by xanthine dehydrogenase (*XDH*) and urate oxidase (*UOX*). *XDH* is not present in yeast species and therefore they import urate, allantoin or allantoate into the cell to use purine derivatives as nitrogen source. The yeast species which do not posses DAL cluster are capable of using urate as a nitrogen source using urate permease (*UAP*) to import it and *UOX* to oxidize it and the enzymes of DAL metabolic pathway to degrade it to urea [32]. *S. cerevisiae* and *N. castellii* do not have *UAP* and *UOX* and therefore are unable to use urate and thus import allantoin as nitrogen source using allantoin permease gene *DAL4*. Once imported aallantoin is then degraded to simpler form of nitrogen using the same DAL pathway genes in all yeasts however, in *S. cerevisiae* and *N. castellii* the DAL genes have been organized into a cluster. The ability to import allantoin instead of urate ruled out the oxygen requiring step performed by *UOX* leading to the biochemical reorganization of purine degradation pathway. The species which possess the DAL cluster have the ability to grow under oxygen limiting conditions [21].

Firstly, we tested the growth of our wild type strain on different concentrations of allantoin and found that a high concentration of 0.4% (w/v) was deleterious to the strain while a concentration of 0.0125% (w/v) was just suboptimal and therefore ideal to detect fitness changes in the engineered mutant strains (Figure S1). Then, we measured the growth rate of the wild type *N. castellii* and *S. cerevisiae* (FY3) in F1 medium and F1 medium containing allantoin as nitrogen source. It was observed that wild type *N. castellii* was growing much slower than *S. cerevisiae* in both allantoin and F1 media (Figure S2). There was a 7% decline of growth rate for *N. castellii* in F1 medium and 68% decline of growth rate in allantoin medium relative to *S. cerevisiae*. These results indicated that *S. cerevisiae* can utilize allantoin in much more growth efficient way than *N. castellii* wild type strain.

### Construction of the *S. cerevisiae* strains with different gene inversions

To study the impact of gene inversions on gene expression, we constructed inverted and non-inverted control strains using the cre-*lox*P system [33]. Wild type *S. cerevisiae* (FY3) was the background strain used to construct all inverted and non-inverted control strains. The non-inverted control strains will be referred as “control” in the text. In total, we constructed two strains with a single gene inversion, one with double gene inversion, and two triple gene inversions along with their respective control strains which were collinear to *S. cerevisiae* but with two *lox*P or *lox*2272 scars at the inversion breakpoints (Figure S3). The lists of all the strains created in the work with the respective inversions are listed in Figure S3.

Compared to *S. cerevisiae*, the three genes *DAL1*, *DAL2* and *DAL4* are inverted in *N. castellii*, with *DAL2* being inverted twice. Since it is not defined knowledge which gene got inverted first we adopted two different strategies to re-create the *N. castellii* like DAL cluster in *S. cerevisiae* background.

**Figure 3:**
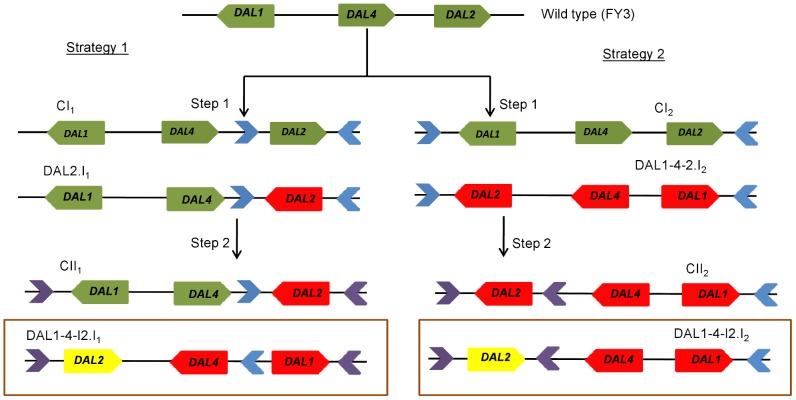
Strategies followed to engineer strains possessing *N. castellii* like DAL cluster. In strategy one the *DAL2* was inverted first followed by the larger inversion of the three genes *DAL1*, *DAL4* and *DAL2*. In strategy two the block of the three genes, *DAL1*, *DAL4* and *DAL2*, was inverted first followed by the single inversion of the *DAL2* gene. The control strains had the *lox*P and *lox*2272 insertions in the intergenic regions, but they do not present the inversion. The red, yellow and green blocks indicate the single inverted, double inverted and non-inverted genes respectively. The *lox*P and *lox*2272 scars are represented as blue and violet triangles respectively.

In the first strategy, the *DAL2* gene was inverted first creating the DAL2.I_1_ strain which was then used to generate the inversion of the DNA sequence containing *DAL1*, *DAL4* and *DAL2*. In the second strategy the DNA segment with *DAL1*, *DAL4* and *DAL2* was inverted first creating the DAL1-4-2.I_2_ strain, which was then used to invert the *DAL2* gene. Both strategies resulted in the construction of a final *S. cerevisiae* strain with the DAL cluster order similar to that of *N. castellii* (DAL1-4-I2.I_1_ and DAL1-4-I2.I_2_) (Figure 3). Control strains were created identical to their parent strains except for the presence of the *loxP and lox*2272 scars (CI_1_, CI_2_, CII_1_ and CII_2_) (Figure 3).

### Fitness assays of the engineered strains in different nitrogen sources

The fitness of inverted and control strains was determined in rich medium (YPD), minimal medium containing allantoin as nitrogen source and F1 medium. We did not observe any significant changes in the growth rate of all the inverted and control strains in YPD and F1 medium.

**Figure 4:**
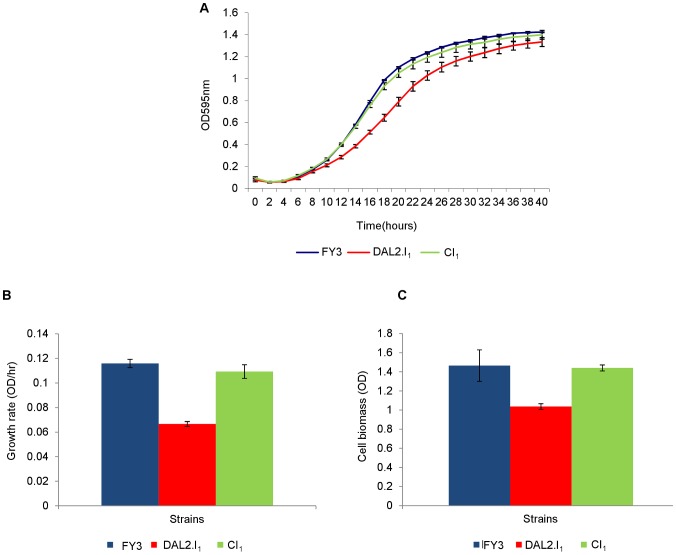
Fitness assay of wild type (FY3) and engineered strains. The growth rate of DAL2.I_1_ strain (red line) was found to be lower than the FY3 (blue line) and control strain (green line) in F1 medium supplemented with 0.0125% (w/v) allantoin. The growth curve was derived from the OD values obtained from plate reader (**A**). The maximum growth rate and cell biomass were calculated using the R statistic package grofit and the results are shown in panel **B** and **C**. The bars represent the mean of three technical replicas of five independent biological replicas for each strain respectively. Error bars are at 95% confidence intervals.

For the strains engineered using the first strategy, we observed that in the allantoin containing medium, the *DAL2* gene inversion affected the growth rate of DAL2.I_1_ in comparison to the FY3 and CI_1_ strains. There was a 38% drop in growth rate for the DAL2.I_1_ strain relative to the CI_1_ and FY3 strains (Figure 4). The same loss of fitness was observed after the inversion of the DNA sequence containing *DAL1*, *DAL4* and *DAL2* in the DAL1-4-I2.I_1_ when compared to FY3 and CI_1_. This result suggested that that the second larger inversion did not contribute any further to the defective phenotype.

Using the inverted strains created with the second strategy the strain DAL1-4-I2.I_2_ with the larger inversion encompassing *DAL1*, *DAL2* and *DAL4* did not show any change in fitness compared to the control and wild type strains in allantoin medium (Figure S4). However, when the second inversion of just *DAL2* was generated in the DAL1-4-I2.I_2_ strain there was a drop in the growth rate of the inverted strains compared to the control and FY3 (Figure S4). Again, these results suggest that the *DAL2* inversion is the main cause of the drop in fitness. The other engineered inverted strains DAL3.I and DAL3-7.I did not show any significant gain or loss in the growth rate (Figure S5).

A recent study showed that clustering of GAL genes in *S. cerevisiae* is not linked to positive fitness effects, since the disruption of GAL gene cluster *(GAL1-GAL10-GAL7)* did not have any phenotypic effect [34]. Our study established that disrupting the gene order of the DAL metabolic cluster has an effect on the growth rate. In particular, the inversion of *DAL2* was responsible of the fitness change.

### Effect of *DAL2* inversion on the expression of DAL cluster genes

We looked at the expression level of the DAL genes between inverted and control strains by performing real-time quantitative PCR. Any change in expression occurring due to insertion of *lox*Ps or *lox*2272s was taken into consideration by normalizing the expression of inverted strains with the control strains. For each sample, the housekeeping *ACT1* was amplified and all data were normalized to *ACT1*.

**Figure 5:**
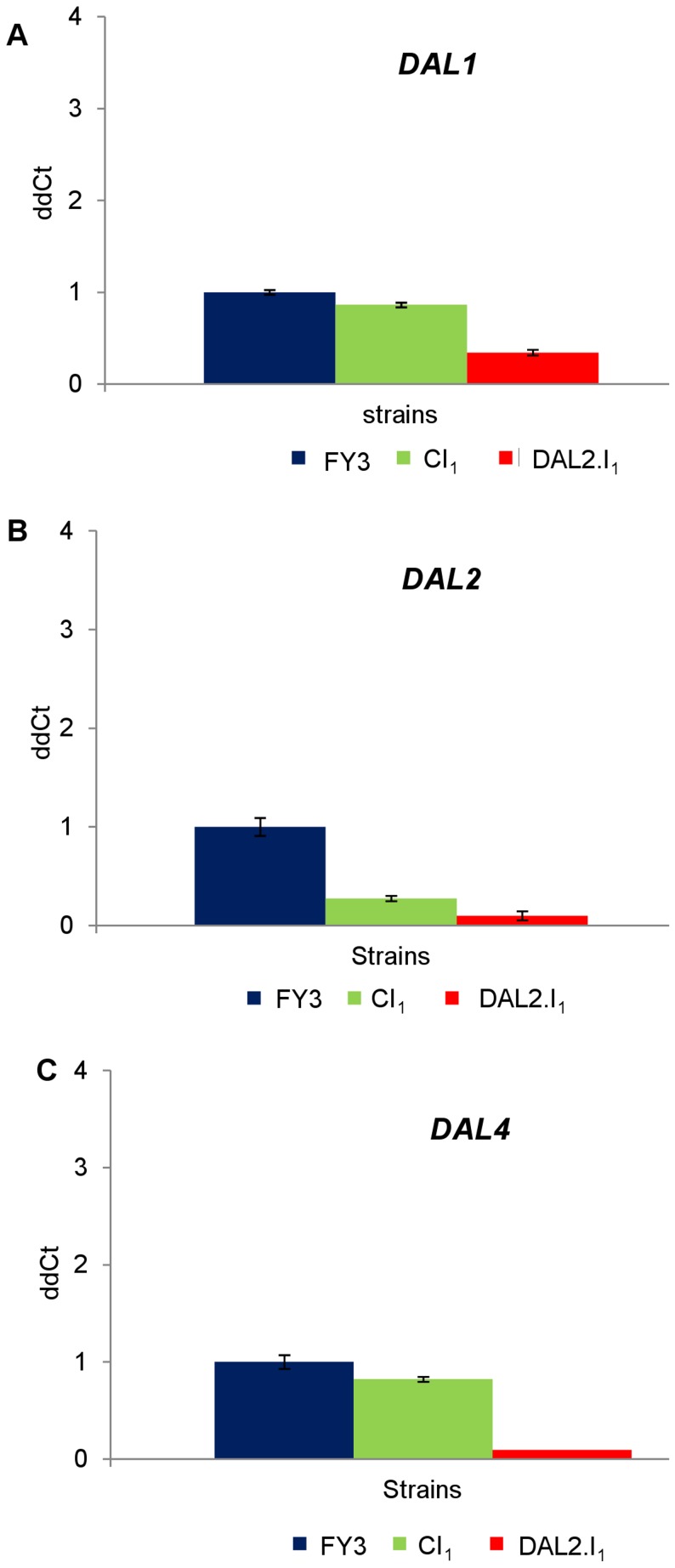
Expression levels of *DAL2*, *DAL4* and *DAL1* genes in the DAL2.I_1_ and CI_1_ strains. Real time PCR to show the expression of *DAL1* (**A**), *DAL2* (**B**) and *DAL4* (**C**). Blue, green and red, boxes represents the FY3 control and inverted strains, respectively. Error bars are from three technical replicas for each of the three independent biological samples. Relative normalized fold expression was calculated by using ΔΔCt method and *ACT1* as a reference gene.

Our fitness assays showed that inversion of *DAL2* reduces the growth rate of inverted strain as compared to the control strain. Our next approach was to study the affect of inversion on *DAL2* expression and also of its neighbouring genes expression such as *DAL1* and *DAL4*. We observed about 50% decline of *DAL1* gene expression in the DAL2.I_1_ strain, while the *DAL2* and *DAL4* expression level was reduced to ca. 90% (Figure 5). The expression of *DAL4* and *DAL1* in control strains was similar to the WT, while the expression of *DAL2* in the control strain was at least 50% lower than the wild type suggesting an effect of the *lox*P insertion in the transcript level. However, since no phenotypic effect was seen in the control strain, the amount of *DAL2* gene product must have been sufficient to grant normal growth.

Both DAL2.I_1_ and DAL1-4-I2.I_2_ has reduced expression of *DAL4* in allantoin containing medium, and the reduced fitness effect could be explained by the down regulation of *DAL4* as this gene is responsible for allantoin uptake by the cell.

Co-expression of genes can be due to the presence of bidirectional promoters [35], such as in the case of *GAL1* and *GAL10* genes in the GAL cluster [34], or by regulatory mechanisms at chromatin level [36]. Eukaryotic genes that are co-regulated in a cluster are usually inter dependent regarding their expression [37], [38], and it has been reported that genomic neighbourhoods can regulate transcript levels, playing an important role in genome evolution [39], [40]. Our results demonstrate that *DAL2* inversion not only affects its own gene expression but also that one of neighbouring genes (Figure 5). Moreover, *DAL4*, *DAL1* and *DCG1* (but not *DAL2*) have an antisense transcript associated with their mRNA which may play a role in their regulation (Figure S6). Interestingly, studies on the impact of neighbourhood continuity on gene expression in engineered inverted and the non-inverted *Drosophila* strains found that no significant changes occurred in the expression of neighbourhood genes, showing that cluster organisation may not be as important as previously thought in maintaining the correct level of mRNAs in the cell [41]. This difference outcome could be due to the fact that the intergenic regions in yeast are very short with a high density of tightly regulated functional units and therefore small changes in the sequence are more likely to cause an alteration of the gene expression compared to *Drosophila*.

To investigate whether the expression of *DAL4* antisense transcript, *SUT614,* has changed upon inversion of *DAL2*, we carried out strand specific Northern Blot analysis.

### Expression analysis of *DAL4* and *SUT614*

Co-expression of closely located genes is not only dependent upon transcription factors or chromatin structure but is also dependent upon bidirectionally active promoters [42]. *Saccharomyces cerevisiae* possess several transcripts which either exist as stable unannotated transcript (SUTs) or are rapidly degraded and are cryptic unstable transcript (CUTs). We used the bidirectional promoter database developed by Xu *et al*, 2009 to find out the antisense transcripts for DAL genes [35]. It was observed that three out of six DAL genes in the cluster possess stable unannotated transcripts which are *SUT195*, *SUT614* and *SUT196*.

**Figure 6:**
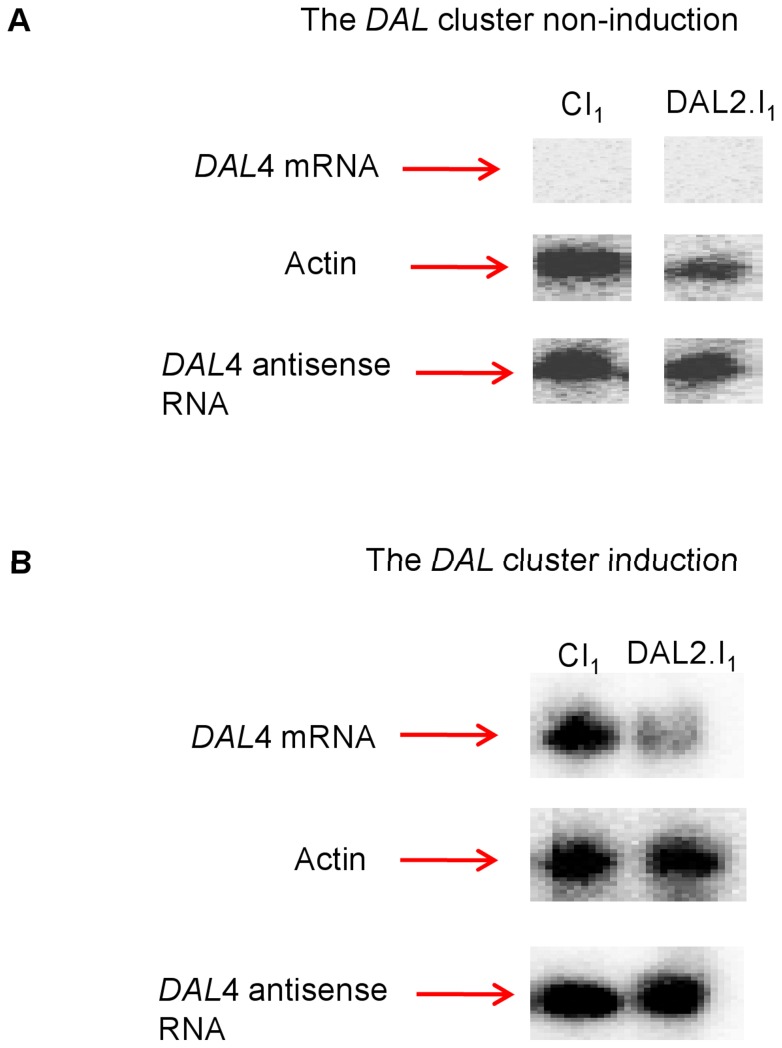
Northern analysis of *DAL4* sense and antisense transcripts. RNA was extracted from cells grown under non-induced conditions (YPD medium, panel **A**) and induced conditions (F1 medium containing Proline+Allantoin as N-source, panel **B**). Oligonucleotides specific for the *DAL4* sense and anti-sense strand were used. As expected no expression of *DAL4* sense transcript was observed in YPD medium while, the antisense *DAL4* signal was very strong (**A**). Under induced conditions the expression of *DAL4* sense was greatly reduced in the inverted strain as compared to the control while the expression of antisense transcript remained same in both strain backgrounds (**B**). *ACT1* was used as the reference gene for expression comparisons.

*SUT614* is the antisense transcript of *DAL4*. To test the role of bidirectional promoter in *DAL4* we looked at the expression of *SUT614* and *DAL4* in our DAL2.I_1_ and control strain. We therefore checked the expression of *DAL4* and *SUT614* in YPD medium and after inducing the DAL cluster in allantoin+proline containing medium. As expected, we observed different levels of expression for *DAL4* and *SUT614* in the two different media. As expected, we observed that in both CI_1_ and DAL2.I1 *DAL4* is not expressed in YPD medium, while the antisense transcript showed a strong expression level (Figure 6A). The expression analysis of *DAL4* and *SUT614* in proline+allantoin medium showed that the *DAL4* expression is reduced in DAL2.I_1_ when compared to its control CI_1_. Moreover, *SUT614* was equally highly expressed in both strains (Figure 6B). This data shows that inverting *DAL2* reduces the expression of its neighboring gene *DAL4* but not that one of the antisense transcript, suggesting that *SUT614* is not responsible for *DAL4* regulation.

## Conclusions and Future Prospects

In this work we re-created in *S. cerevisiae* the two nested inversions present in *N. castellii* DAL cluster, and we showed that the inversion of *DAL2* gene alone was sufficient to impair the growth rate of this yeast when allantoin is given as source of nitrogen. Further studies on the effect of the *DAL 2* inversion in *N. castellii* background would be interesting to understand if the change in the gene order has an impact in the allantoin utilisation in this species as well.

In *S. cerevisiae*, the inverted DAL2.I1 strain affects its own gene expression and that of the neighbouring genes *DAL1* and *DAL4*.

Since *DAL2* inversion is changing the expression of *DAL4* and *DAL1*, it is possible that DAL metabolic pathway is regulated by a feed-back mechanism involving the *DAL2* product. Allantoin permease encoded by *DAL4* is responsible for entry of allantoin into the cell, which is converted to allantoate with help of enzyme allantoinase encoded by *DAL1* gene. *DAL2* encodes for allantoicase and is the main central gene responsible for converting allantoate to simpler form of nitrogen through series of steps. Therefore, it is plausible that the impairment of *DAL2* expression is affecting the whole DAL metabolic pathway and that with the down regulation of *DAL4* the cell is unable to take-in sufficient allantoin for normal growth.

We showed that the inversion of *DAL2* does not affect the expression of “*SUT614*”, although the *DAL4* transcription is significantly compromised. It is therefore unlikely that *DAL4* is regulated by its antisense transcript *SUT614*.

The DAL cluster is sensitive to nitrogen catabolite repression (NCR) and is expressed in the lack of readily available nitrogen sources. Its regulation occurs via three types of cis-acting and trans-acting factors: the UAS_NTR_ binding either Gln3p or Gat1p [43], present upstream of all allantoin pathway genes, the URS_GATA_, associated with DAL80p [44] which is involved in the down-regulation of DAL gene expression, and the UIS_ALL_ binding DAL81p and DAL82p [45]. As summarised in Figure S6, *DAL1*, *DAL2* and *DAL4* require functional *GLN3*, *DAL82* and *DAL81* for transcription. Although the molecular modifications carried out to construct the inverted and control strains did not disrupt specifically the transcription factors binding sites, it is possible that a change of DNA sequence in this location may hamper the ability of these transcription factors to dock on the DNA.

Moreover, the sub-telomeric DAL cluster is placed within Htz1- activated domain (HZAD) which consists of histone H2A variant H2A.Z (Htz1). H2A.Z is present in region of *DAL1*, *DAL2*, *DCG1* and *DAL3* genes of DAL metabolic cluster. It is thought to act as an anti-silencing factor by preventing the spread of Sir protein and therefore activating the expression of these genes. Again it is plausible that the inversion of *DAL2* might have affected the location of H2A.Z histone which in turn altered the expression of these genes.

## Supporting Information

Figure S1 Fitness assay to optimise allantoin concentration. Growth rate of a wild type *S. cerevisiae* strain, FY3, was measured in different concentrations of allantoin containing medium over the course of 40 hours. Higher concentration of allantoin 0.4% (w/v) (blue line), was found to be toxic to the cells. For the fitness assays a sub-optimal concentration of 0.0125% (w/v) of allantoin (red line) was used. Each point represents the mean average of 5 technical replicas. Error bars at 95% confidence interval.
(TIF)

Figure S2 Fitness assay of *N. castellii* (WT) and FY3 in F1 and F1+allantoin medium. The growth profiles of *S. cerevisiae* and *N. castellii* was measured in F1 and F1+allantoin medium. *N. castellii* (red line) is less fit than *S. cerevisiae* (blue line) in both media (**A**). The maximum growth rate and cell biomass were calculated using the R statistic package grofit (**B**). Each point represents the mean average of three technical replicas for five independent biological samples. Error bars are at 95% confidence intervals.
(TIF)

Figure S3 The DAL cluster structure of wild type, inverted and control strains. The *loxP* sequences were inserted in FY3 strain at the inversion breakpoints to construct the single, double and triple inverted strains using the cre-*lox*P system (**A**). The control strains without the inversions but carrying the *lox*P insertions are shown in panel **B**. The red and green blocks indicate the inverted and collinear genes, respectively, whereas the blue triangles represent the *lox*P scars.
(TIF)

Figure S4 Fitness assay of wild type (FY3) and engineered strains. The growth rate of DAL1-4-I2.I_2_ strain (red line), FY3 (blue line) and control strain (green line) in F1 medium supplemented with 0.0125% (w/v) allantoin was found to be the same (**A**). The inversion of *DAL2* in the DAL1-4-I2.I_2_ strain showed a drop in the growth rate of the inverted strains (yellow line) compared to the control strains (B). The growth curves were derived from the OD values obtained from plate reader. The error bars represent the mean of three technical replicas of five independent biological replicas for each strain respectively. Error bars are at 95% confidence intervals.
(TIF)

Figure S5 Fitness assay of DAL3.I and DAL3-7.I along with their respective control strains. The growth rate of DAL3.I strain (red line), FY3 (blue line) and control strain (green line) in F1 medium supplemented with 0.0125% (w/v) allantoin was found to be the same (**A**). The DAL3-7.I inverted strain (red line) also possessed equal growth rate as compared to the FY3 (blue line) and control strain (green line). The growth curves were derived from the OD values obtained from plate reader. The error bars represent the mean of three technical replicas of five independent biological replicas for each strain respectively. Error bars are at 95% confidence intervals.
(TIF)

Figure S6 The map of the trancription binding site and antisense transcript in the DAL cluster. Representation of the DAL genes sense transcripts (green arrows), antisense transcripts (blue arrows), sites of *lox*P insertions (red arrows) and transcription factor binding sites (black arrows).
(TIF)

Table S1 List of checking primers used for confirming inverted and non-inverted strains.
(DOC)

Table S2 List of primers used for real time PCR.
(DOC)

Table S3 List of probes used for northern blotting.
(DOC)

Table S4 The set of cassette amplifying primers for engineering inverted and non-inverted strains.
(DOC)

Table S5 The genotypes of *Saccharomyces cerevisiae* inverted and non-inverted strains used in this study.
(DOC)
