# Supplementary figures and images for: Impact of Chromosomal Inversions on the Yeast DAL Cluster
Source: PLoS One. 2012 Aug 14;7(8):e42022. doi: 10.1371/journal.pone.0042022 (PMC3419248; doi:10.1371/journal.pone.0042022)

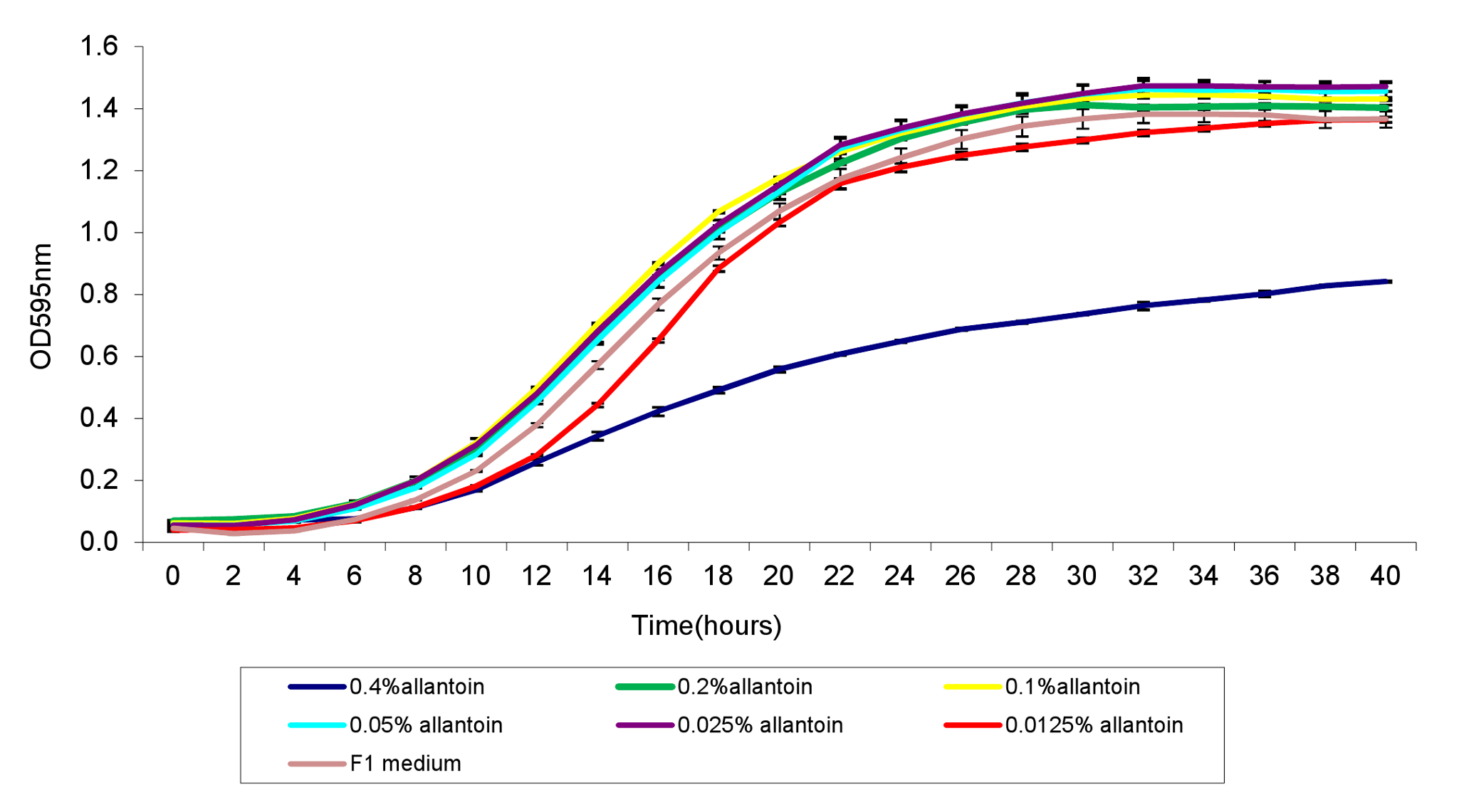

Supplement: Figure S1 — Fitness assay to optimise allantoin concentration. Growth rate of a wild type S. cerevisiae strain, FY3, was measured in different concentrations of allantoin containing medium over the course of 40 hours. Higher concentration of allantoin 0.4% (w/v) (blue line), was found to be toxic to the cells. For the fitness assays a sub-optimal concentration of 0.0125% (w/v) of allantoin (red line) was used. Each point represents the mean average of 5 technical replicas. Error bars at 95% confidence interval. (TIF) [file pone.0042022.s001.tif]

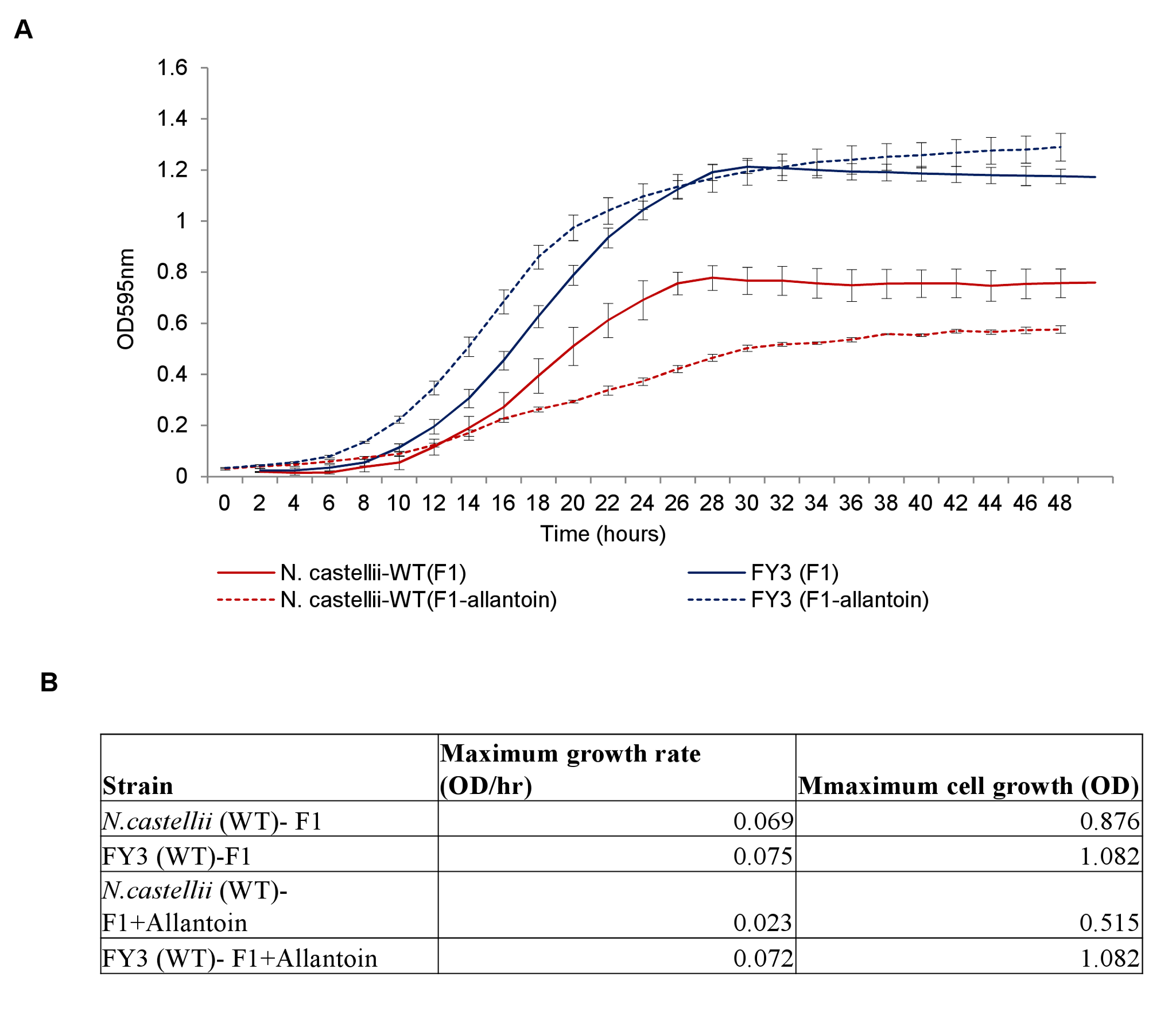

Supplement: Figure S2 — Fitness assay of N. castellii (WT) and FY3 in F1 and F1+allantoin medium. The growth profiles of S. cerevisiae and N. castellii was measured in F1 and F1+allantoin medium. N. castellii (red line) is less fit than S. cerevisiae (blue line) in both media (A). The maximum growth rate and cell biomass were calculated using the R statistic package grofit (B). Each point represents the mean average of three technical replicas for five independent biological samples. Error bars are at 95% confidence intervals. (TIF) [file pone.0042022.s002.tif]

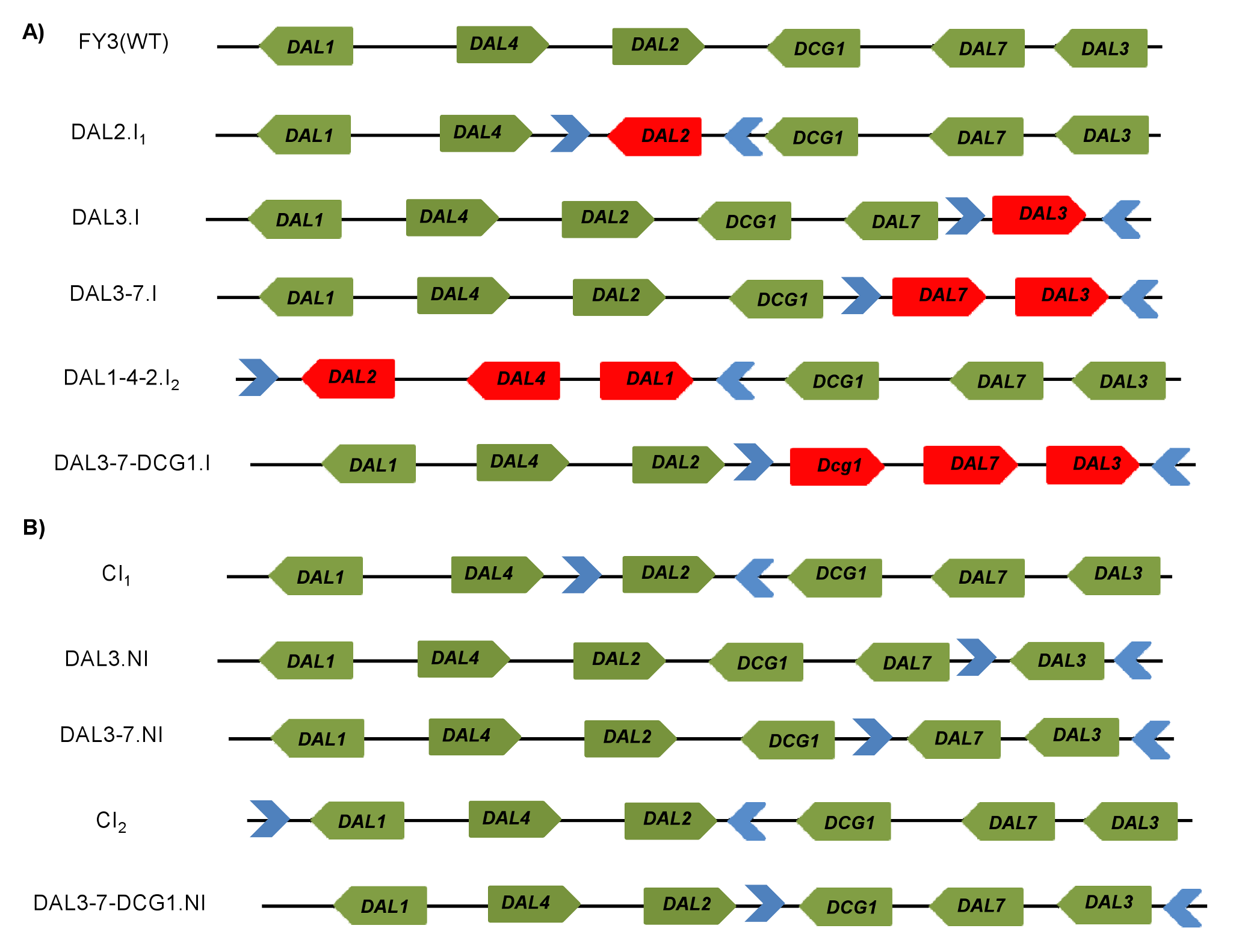

Supplement: Figure S3 — The DAL cluster structure of wild type, inverted and control strains. The loxP sequences were inserted in FY3 strain at the inversion breakpoints to construct the single, double and triple inverted strains using the cre-loxP system (A). The control strains without the inversions but carrying the loxP insertions are shown in panel B. The red and green blocks indicate the inverted and collinear genes, respectively, whereas the blue triangles represent the loxP scars. (TIF) [file pone.0042022.s003.tif]

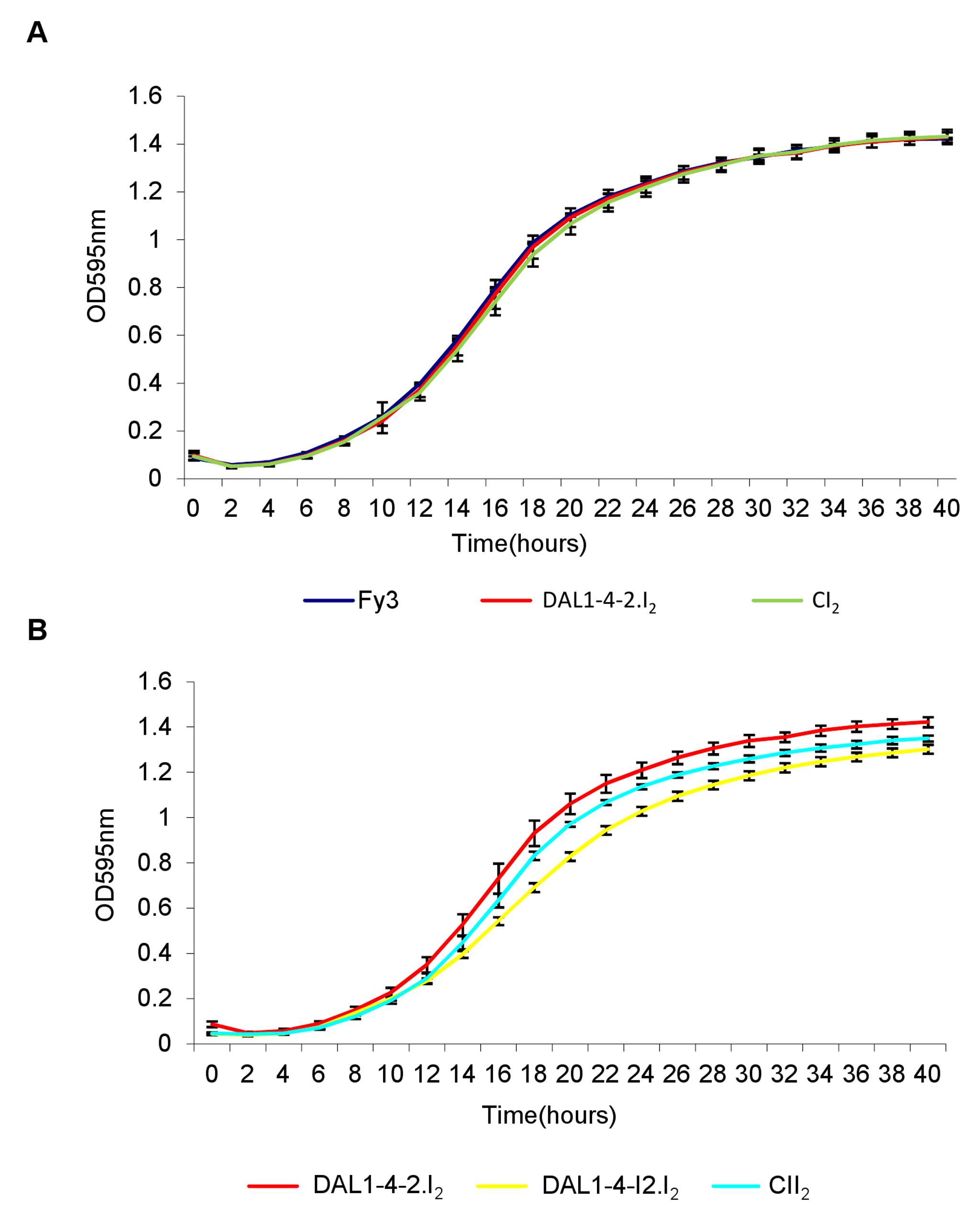

Supplement: Figure S4 — Fitness assay of wild type (FY3) and engineered strains. The growth rate of DAL1-4-I2.I2 strain (red line), FY3 (blue line) and control strain (green line) in F1 medium supplemented with 0.0125% (w/v) allantoin was found to be the same (A). The inversion of DAL2 in the DAL1-4-I2.I2 strain showed a drop in the growth rate of the inverted strains (yellow line) compared to the control strains (B). The growth curves were derived from the OD values obtained from plate reader. The error bars represent the mean of three technical replicas of five independent biological replicas for each strain respectively. Error bars are at 95% confidence intervals. (TIF) [file pone.0042022.s004.tif]

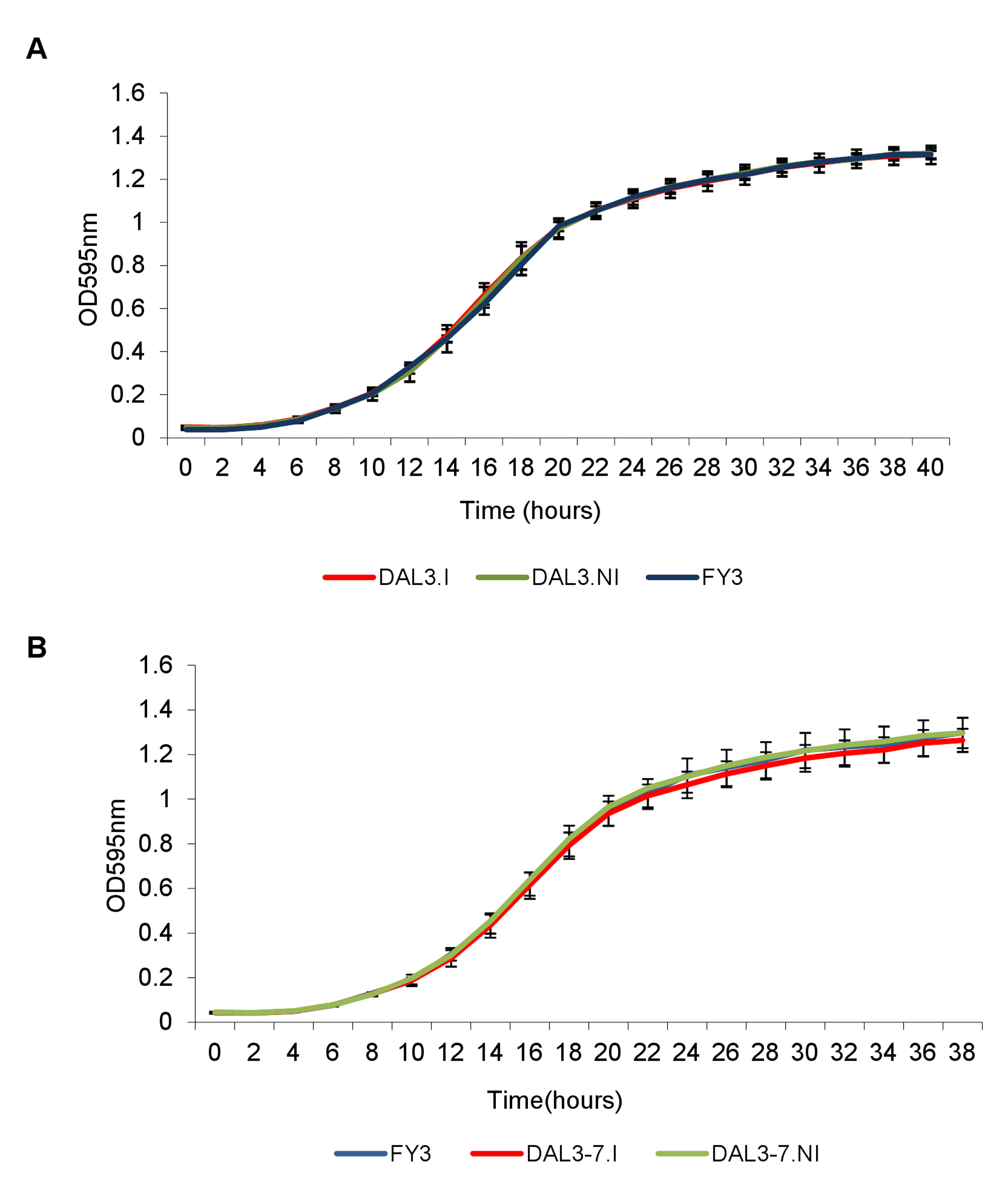

Supplement: Figure S5 — Fitness assay of DAL3.I and DAL3-7.I along with their respective control strains. The growth rate of DAL3.I strain (red line), FY3 (blue line) and control strain (green line) in F1 medium supplemented with 0.0125% (w/v) allantoin was found to be the same (A). The DAL3-7.I inverted strain (red line) also possessed equal growth rate as compared to the FY3 (blue line) and control strain (green line). The growth curves were derived from the OD values obtained from plate reader. The error bars represent the mean of three technical replicas of five independent biological replicas for each strain respectively. Error bars are at 95% confidence intervals. (TIF) [file pone.0042022.s005.tif]

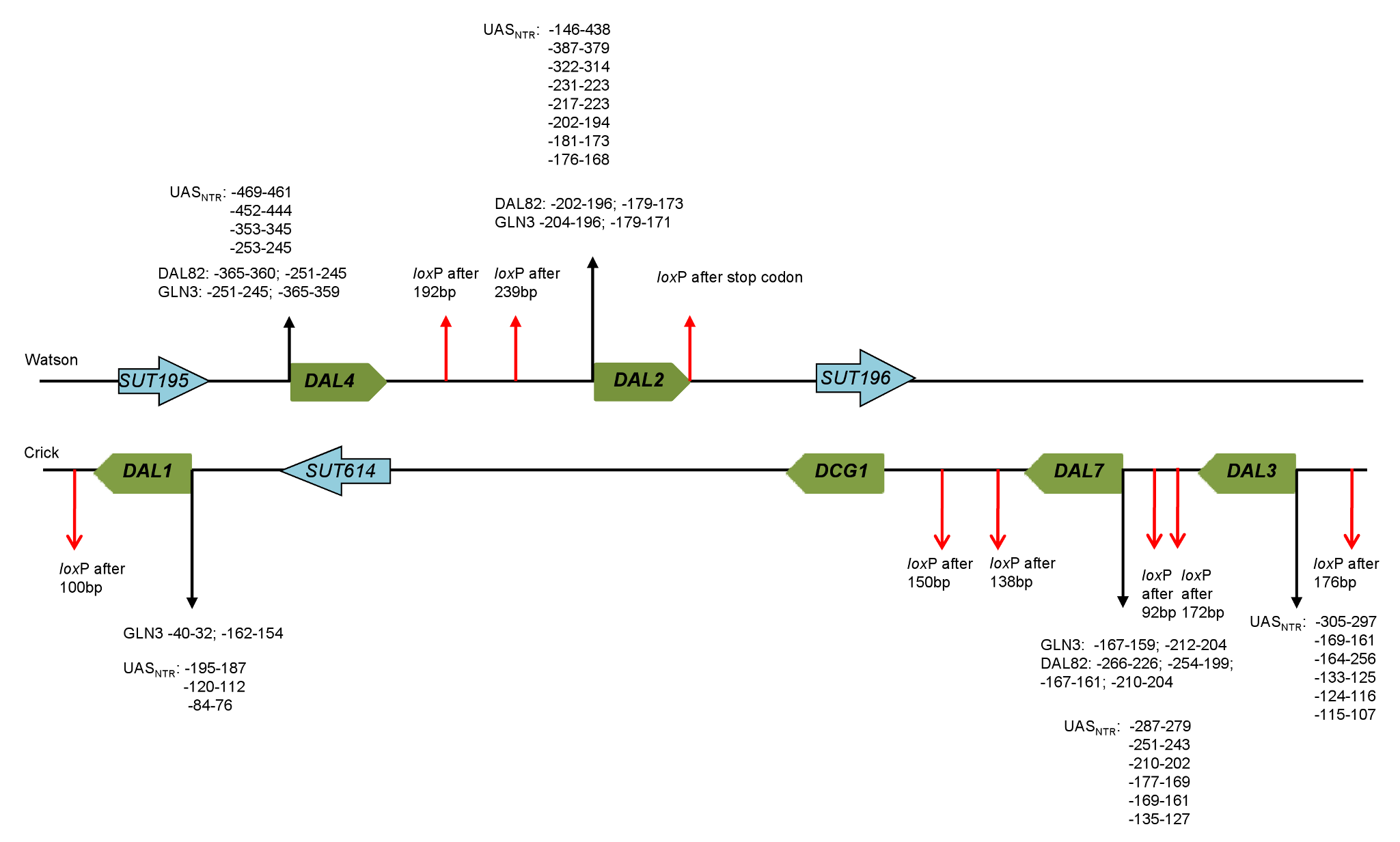

Supplement: Figure S6 — The map of the trancription binding site and antisense transcript in the DAL cluster. Representation of the DAL genes sense transcripts (green arrows), antisense transcripts (blue arrows), sites of loxP insertions (red arrows) and transcription factor binding sites (black arrows). (TIF) [file pone.0042022.s006.tif]
