# Supplementary material for: Impact of Chromosomal Inversions on the Yeast DAL Cluster
Source: PLoS One. 2012 Aug 14;7(8):e42022. doi: 10.1371/journal.pone.0042022 (PMC3419248; doi:10.1371/journal.pone.0042022)
Supplement: Table S1 — List of checking primers used for confirming inverted and non-inverted strains. (DOC) [file pone.0042022.s007.doc]

**Table S1. List of checking primers used for confirming inverted and non-inverted strains**

| **Primer Name** | **Sequence 5'-3'** | **Tm (°C)** |
| --- | --- | --- |
| DAL1_FC | CACCCATCCGCTCTGAGT | 58.2 |
| DAL1_RC | AATTGCAGCCGTGGAGAC | 56 |
| DCG1_RC | GGATGGCACG CTGTTTCT | 56 |
| Dal2_FC | GCTCTATGACGAGGGCGAAG AAG | 59.4 |
| hph-up | AGTTCGGTT TCAGGCAGGT | 56.7 |
| hph-down | AGCATCAGCTCATC GAGAG | 56.7 |
| DAL4.F | CTGTCCCTGGTACTCCCGTA | 61.4 |
| DAL2.R | CTATGACGAGGGCGAAGA AG | 59.4 |
| P3K4up | AACGTGAGTCTTTTCCTTACC | 60.1 |
| P4K4down | TCTCCTTCATTACAGAAACG | 57.3 |
| nat_down | GCTGACCGTCGAGGACAT | 58.2 |
| nat_up | ATGTCCTCGACGGTCAGC | 58.2 |
| D1 | CCCCAAGAACGACTGTTGAT | 57 |
| D2 | AGCAATTTGATCGCTGTTCC | 55 |
| D3 | TGCGCCCATGATAGTACTTG | 57 |
| D4 | TTGAGGAGTCGTGTCGATGT | 57 |
| D5 | TGGTTAAGGGGATCTGGTTG | 57 |
| D6 | GGGAAGACAGGCCTCCATAC | 61 |
| D7 | GGATGGCACGCTGTTTCT | 56 |
| D8 | AACTGGGCAGGGAGTCTTCT | 59 |
