# Supplementary material for: Impact of Chromosomal Inversions on the Yeast DAL Cluster
Source: PLoS One. 2012 Aug 14;7(8):e42022. doi: 10.1371/journal.pone.0042022 (PMC3419248; doi:10.1371/journal.pone.0042022)
Supplement: Table S2 — List of primers used for real time PCR. (DOC) [file pone.0042022.s008.doc]

**Table S2. List of primers used for real time PCR.**

| **Primer Name** | **Sequence 5'-3'** | **Tm (°C)** |
| --- | --- | --- |
| RT-DAL1_R | GTCATCCCGTTGTAGGCAGT | 59.4 |
| RT-DAL1_F | AGGCTGCTCTTTGGTCGATA | 57.3 |
| RT-DAL2_R | CCCCGTCTTTGACTTACCAA | 57.3 |
| RT-DAL2_F | CATTACCGTGGAAGGTTGCT | 57.3 |
| RT-DAL4_F | GGGCTGGTGTGTTCTTGATT | 57.3 |
| RT-DAL4_R | CTTGGAAACAAAGCCGTCAT | 55.3 |
| RT-DCG1_F | TCGAAGTCAATGACGGTGTC | 57.3 |
| RT-DCG1_R | CTTGTTTCCTGCCCATCAAT | 55.3 |
