# Supplementary material for: Impact of Chromosomal Inversions on the Yeast DAL Cluster
Source: PLoS One. 2012 Aug 14;7(8):e42022. doi: 10.1371/journal.pone.0042022 (PMC3419248; doi:10.1371/journal.pone.0042022)
Supplement: Table S3 — List of probes used for northern blotting. (DOC) [file pone.0042022.s009.doc]

**Table S3. List of probes used for northern blotting.**

| **Probes** | **Sequence 5'-3'** | **Tm (°C)** |
| --- | --- | --- |
| DAL4-sense1 | ATAGTATGGTATGCCGTGCAAGCCTGGTTAGGTGCAACGC | 74.6 |
| DAL4-antisense1 | GCGTTGCACCTAACCAGGCTTGCACGGCATACCATACTAT | 74.6 |
| New DAL4-sense2 | TGTCCAACCCTCCACATCTATTGTGTCATATACA | 67.1 |
| New DAL4-antis2 | TGTATATGACACAATAGATGTGGAGGGTTGGACA | 67.1 |
| New DAL4-sense3 | GTCCTGGTTCAATTACTTATATTTCTGGCTGGCA | 67.1 |
| New DAL4-antis3 | TGCCAGCCAGAAATATAAGTAATTGAACCAGGAC | 67.1 |
| New DAL4-sense4 | AAGATTGGAAGAATGAGTTACGTAGGGATGACCT | 67.1 |
| New DAL4-antis4 | AGGTCATCCCTACGTAACTCATTCTTCCAATCTT | 67.1 |
| New DAL4-sense5 | TGGAGACCACTTACACCAGAGGTACTAGGGCTGGTGTGTT | 74.6 |
| New DAL4-antis5 | AACACACCAGCCCTAGTACCTCTGGTGTAAGTGGTCTCCA | 74.6 |
| New Actin-sense1 | ATCTATCGTCGGTAGACCAAGACACCAAGGTA | 68.2 |
| New Actin-antis1 | TACCTTGGTGTCTTGGTCTACCGACGATAGAT | 68.2 |
| New Actin-sense2 | AACTTTCAACGTTCCAGCCTTCTACGTTTCCAT | 67 |
| New Actin-antis2 | ATGGAAACGTAGAAGGCTGGAACGTTGAAAGTT | 67 |
| New Actin-sense3 | GCTCCATCTTCCATGAAGGTCAAGATCATTGCTCCTCCAG | 73.6 |
| New Actin-antis3 | AGCAATGATCTTGACCTTCATGGAAGATGGAGC | 68.2 |
