# Supplementary material for: Impact of Chromosomal Inversions on the Yeast DAL Cluster
Source: PLoS One. 2012 Aug 14;7(8):e42022. doi: 10.1371/journal.pone.0042022 (PMC3419248; doi:10.1371/journal.pone.0042022)
Supplement: Table S4 — The set of cassette amplifying primers for engineering inverted and non-inverted strains. (DOC) [file pone.0042022.s010.doc]

**Table S4. The set of cassette amplifying primers for engineering inverted and non-inverted strains**

| **Primer Name** | **Sequence 5'-3'** | **Tm(°C)** |
| --- | --- | --- |
| Dal1_F | AACACCATTGGGTCAAACTTTGCTTGATTCTAGACGTTAAcactatagggagaccggcag | >75 |
| Dal1_R | TTTCTATAGAATTTCTTTAATAAAAATTTGCAACTTTAGTcgtacgctgcaggtcgac | 71.5 |
| DAL2_SN_(F) | CCAGATGGAGGAGTGAAAAGAATAAGAGTTTGGGGGTACTGAcactatagggagaccggcaga | >75 |
| DAL2_SN_(R) | AGAGTGCATTGGTTTAAAATATACAGTAGTTAAGTATTTATCAGTACCCCCAAACTCT cgtacgctgcaggtcgac | >75 |
| ChIXDAL4/2ins.F | GTACTGATGATTAGCTAAACCGTCATCAACCTACCAAACTTGCGC cgtacgctgcaggtcgac | 72.8 |
| ChIXDAL4/2ins.R | GTCAAAGATAAGATGTCGGAATTATCCGGAGTTCTGATAGGCTC cactatagggagaccggcag | 72.8 |
| lox2272_F | GTCAAAGATAAGATGTCGGAATTATCCGGAGTTCTGATAGGCTC cactatagggagaccggcaga | >75 |
| lox2272_R | AGAGTGCATTGGTTTAAAATATACAGTAGTTAAGTATTTATCAGTACCCCCAAACTCT cgtacgctgcaggtcgac | >75 |
| DAL1+ lox2272 | TTTCTATAGAATTTCTTTAATAAAAATTTGCAACTTTAGTcgtacgctgcaggtcgacgg | 72.8 |
