# Supplementary material for: Impact of Chromosomal Inversions on the Yeast DAL Cluster
Source: PLoS One. 2012 Aug 14;7(8):e42022. doi: 10.1371/journal.pone.0042022 (PMC3419248; doi:10.1371/journal.pone.0042022)
Supplement: Table S5 — The genotypes of Saccharomyces cerevisiae inverted and non-inverted strains used in this study. (DOC) [file pone.0042022.s011.doc]

**Table S5. The genotypes of *Saccharomyces* *cerevisiae* inverted and non-inverted strains used in this study.**

| **Strains** | **Genotype** | **Source** |
| --- | --- | --- |
| FY3 (*S. cerevisiae*) | *MATa ura3-52* | Brachmann *et* *al*., 1998 |
| DAL2.I_1_ | FY3 p*DAL2*:: *loxP* + t*DAL2*::*loxP* (inverted) | This study |
| CI_1_ | FY3 p*DAL2*:: *loxP* + t*DAL2*::*loxP* (non-inverted) | This study |
| DAL3.I | FY3 p*DAL3*:: *loxP* + t*DAL3*::*loxP* (inverted) | This study |
| DAL3.NI | FY3 p*DAL3*:: *loxP* + t*DAL3*::*loxP* (non-inverted) | This study |
| DAL3-7.I | FY3 t*DAL3*:: *loxP* + p*DAL7*::*loxP* (inverted) | This study |
| DAL3-7.NI | FY3 t*DAL3*:: *loxP* + p*DAL7*::*loxP* (non-inverted) | This study |
| DAL1-4-2-I_2_ | FY3 t*DAL1*:: *loxP* + t*DAL2*::*loxP* (inverted) | This study |
| CI_2_ | FY3 t*DAL1*:: *loxP* + t*DAL2*::*loxP* (non-inverted) | This study |
| DAL3-7-DCG1.I | FY3 t*DAL3*:: *loxP* + p*DCG1*::*loxP* (inverted) | This study |
| DAL3-7-DCG1.NI | FY3 t*DAL3*:: *loxP* + p*DCG1*::*loxP* (non-inverted) | This study |
| DAL1-4-I2.I_1_ | FY3 t*DAL1*:: *loxP* + p*DAL2*::*lox2272 + tDAL2::lox2272* (inverted) | This study |
| CII_1_ | FY3 t*DAL1*:: *loxP* + p*DAL2*::*lox2272 + tDAL2::lox2272* (non-inverted) | This study |
| DAL1-4-I2.I_2_ | FY3 t*DAL1*:: *loxP* + p*DAL2*::*lox2272 + tDAL2::loxP* (inverted) | This study |
| CII_2_ | FY3 t*DAL1*:: *loxP* + p*DAL2*::*lox2272 + tDAL2::loxP* (non-inverted) | This study |
